# Supply chain contagion and the role of industrial policy

**DOI:** 10.1007/s40812-020-00167-6

**Published:** 2020-07-14

**Authors:** Andrea Coveri, Claudio Cozza, Leopoldo Nascia, Antonello Zanfei

**Affiliations:** 1 Department of Economics, Society and Politics, University of Urbino, Urbino, Italy; 2 Department of Economics and Legal Studies, University of Naples “Parthenope”, Naples, Italy; 3 ISTAT, Rome, Italy

**Keywords:** COVID-19, Pandemic, Contagion, Global value chains, Industrial policy, F12, F60, O25

## Abstract

The COVID-19 pandemic triggered a major disruption in global value chains (GVCs) that pushed the global economy into a recession that promises to be worse than the 2008 crisis. This article illustrates the mechanisms through which the COVID-19 pandemic affected GVCs in the context of a changing configuration of the global economy. In particular, it is argued that GVCs became the main transmission channels of “economic contagion”. Finally, we posit that the pandemic provides an opportunity to revive the role of industrial policy as to govern the landslides of a world economy constantly pressured by globalization and deglobalization forces.

## Introduction

The diffusion of COVID-19 represents the most serious pandemic since the Spanish flu over the 1918–1920 period. After the outbreak in China in the second half of February 2020, the pandemic spread worldwide in a few weeks. The large lockdown of production that followed has immediately shown its disruptive impact and promises to trigger the most serious economic crisis since the Great Recession of 2008. Among the most affected countries are in fact the largest industrialized economies in the world, primarily the United States, Spain, Italy, Germany, the United Kingdom and France, as well as China itself and Japan, responsible overall for over 60% of global GDP (Baldwin and Tomiura 2020).

This short contribution illustrates the impact of the COVID-19 pandemic on the current configuration of global economy (Sect. 2), stressing the role of global value chains (GVCs) as the main transmission channels of “economic contagion” worldwide (Sect. 3). In this context, we provide a focus on Italy, which has been the first economy to be affected in Europe, and most substantially. Section 4 highlights the challenges imposed by the pandemic and discusses the key role that industrial policy should play to set the global economy on a more resilient and sustainable path. Finally, the last section draws some conclusions.^1^

## Supply- and demand-side contagion channels

COVID-19 pandemic occurs in the midst of a transformation that has been characterizing the world economy over the last four decades. Since the 1980s, a series of technological, political and institutional factors gave rise to what Baldwin (2016) called the “2nd unbundling”, meaning a powerful push towards the geographical dispersion of value-added tasks required to get the realization of final products. This vertical fragmentation of production on a global scale has given rise to global value chains (GVCs), along which East Asia and China in particular represent a crucial node (Baldwin 2013; Gereffi and Fernandez-Stark 2016; Zanfei et al. 2019). In other words, the sophisticated *interconnection* of the productive structures of the different countries at the international level led the *interdependence* of the economies to increase remarkably. As a consequence, a production bottleneck in a hub of the dislocated supply chain can trigger a synchronized economic slowdown and, therefore, a global downturn (Baldwin and Weder di Mauro 2020; Boehm et al. 2019; Inoue and Todo 2020; Sforza and Steininger 2020; UNCTAD 2020).

Therefore, the *first channel* through which the pandemic has affected the global economy concerns the interruption of the supply chains, hitting what has been identified as the productive heart of the world, that is East Asia, and China in particular (Buckley and Strange 2015). In this regard, consider that Chinese industrial production has fallen by 13.5% on an annual basis between January and February 2020, i.e. the biggest drop in production in China since the post-Deng Xiaoping era, especially in the transport equipment (− 28.2%), general equipment (− 28.2%), textiles (− 27.2%) and machinery (− 24.7%) (data from National Bureau of Statistics of China).

The *second, and related, channel* concerns the amplification effect of the contagion induced by the chains of global subcontracting of intermediate goods, since even the manufacturing sectors of the least affected countries have had difficulty in acquiring (importing) the intermediate inputs necessary for domestic production (Baldwin and Tomiura 2020; Bonadio et al. 2020). In this context, the loosening of the production lockdown in China (in March), while it was being strengthened in Europe and the United States (in April), adds further complexity to this scenario. On the one hand, the attempts of Western importing companies to reduce their dependence on intermediate goods from China, the so-called *decoupling*, might turn out a bad strategy, as China is now recovering faster than the rest of world. On the other hand, the global spread of COVID-19 is ultimately harmful also for China, as it imports a large part of the intermediate goods necessary for production for its domestic market, as well as for foreign markets (World Bank 2020).

In addition, the *demand-side of the GVCs-pandemic nexus* should not be underestimated. Firstly, governments’ measures aimed at containing the virus, such as the drastic reduction of people mobility and the shutdown of almost all commercial and leisure activities, entail an immediate reduction of consumptions. Some of them will be postponed but many others will be probably never recovered. The heaviest effects will concern the services sector, in particular transport, tourism, accommodation and catering sector, thus affecting some countries (e.g. Italy) more than others. Secondly, the sharp slowdown in production is expected to generate an increase in the unemployment rate, which will result in a reduction in households’ disposable income, starting with those who are hired on a temporary basis. If this reduction mainly affects the poorer segments of the population—those with a higher marginal propensity to consume—the consequences on aggregate consumption will be even more significant. In addition, due to the uncertainty caused by the speed and spread of the infection, it is reasonable to expect a reduction in investment rates and an increase in precautionary saving as a form of protection for a dark future. Thirdly, a lower utilization rate of production capacity by firms could make it more difficult for them to amortize fixed costs (a trivial example is the rental cost of buildings). This in turn could lead to an increase in unit costs, a reduction in the profit rates and therefore a further contraction in investment expenditure. Finally, the global reduction in consumption and investments amplifies the value added contraction simultaneously, further restricting foreign outlet markets and therefore slowing down the dynamics of net exports of both final and intermediate goods.

Although our focus is on economic consequences of the pandemic, it might be worth mentioning an important feedback effect. In fact, COVID-19 may not only affect GVCs, but its diffusion might have been favored by the increasing resort to international production networks. One often emphasized mechanism is through increase in pollution. Some scientists suggest that industrial production is altering ecosystems and, above all, reducing biodiversity (Wallace 2016). This reduction would result in a greater probability of the cross-species transmission of viruses. However, the probability of virus transmission is not only likely to be connected to industrial production in general, but more specifically to the modern hyper-globalized world economy.

This link from GVCs to COVID-19 holds for at least two reasons. First, consider the extent to which the GVCs have increased the concentration of manufacturing production in certain geographical areas, particularly in East Asia but also in Latin America. This concentration is accompanied by an accentuated specialization of such regions in the most energy-intensive manufacturing stages of production, taking advantage of cost reductions with little consideration of environmental issues. This is often due to the very low cost of local labor, which in turn depends on the large pool of workers in emerging countries and the less restrictive legislation compared to western economies. Environmental regulation is also less restrictive than in other parts of the world, attracting polluting manufacturing activities which could contribute to increasing the likelihood of new viruses.

Second, the logic of GVCs meant a transnational dislocation of production which has at least partially required greater workers’ mobility. Given that GVCs are dominated by global actors, the international mobility of their workers, especially managers of multinational corporations (MNCs), has likely played a role on the spread of the virus. Although anecdotal, the first detected cases of “patient one” in Germany, a manager infected by a colleague from Shanghai who allegedly brought the virus to Germany on January 20, 2020 (Rothe et al. 2020); or the circumstance that the first case in the Italian village of Codogno was a manager of Unilever, are emblematic.

## Global economic structure and the impact on Italy

**Fig. 1:**
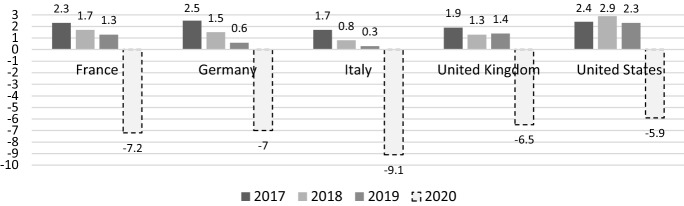
Real GDP growth (annual % change), selected countries, 2017–2020. Source: authors’ elaboration on IMF data. Note: the 2020 growth rates are projections provided by IMF (2020)

The shock caused by COVID-19 pandemic occurred at a time when the global economy was already on the verge of a recession. Figure 1 reports the progressive reduction in the GDP growth rates of major economic regions—especially European ones—from 2017 to 2019 along with the IMF growth projection to 2020 (IMF 2020). According to the latter, the outbreak of the pandemic will lead to a global GDP contraction of 3% for 2020. Notably, the world GDP contraction during the Great Recession in 2009 was equal to 0.1%.

With regard to the European Union, IMF (2020) estimates a 7.1% reduction in GDP, with Italy called to suffer the biggest backlash: a 9.1% decrease with respect to the previous year. To fully acknowledge the magnitude, consider that in 2009, i.e. the worst year of the past global financial crisis, Italy's GDP fell by just under 6% and at that time the unemployment rate in Italy soared to almost 13% at the end of 2014 (data from OECD).

**Fig. 2:**
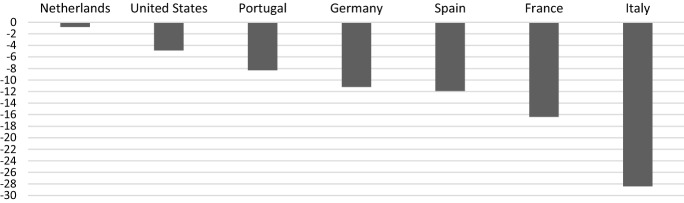
Industrial production growth in March 2020 (monthly basis, % change), selected countries. Source: authors’ elaboration on Eurostat data for EU countries and tradingeconomics.com for the US

Moreover, preliminary data provided by the Italian National Institute of Statistics (ISTAT) show a decrease of 28.4% in industrial production in March 2020 as compared to February for Italy—much worse than in other industrialized countries, as reported in Fig. 2—with a drop of 57.1% in durable consumption goods and of 39.9% in capital goods on a yearly basis. In addition, preliminary estimations provided by Prometeia report that the pandemic would lead to an overall reduction of the Italian industrial production for March and April equal to 61%^2^.

**Fig. 3:**
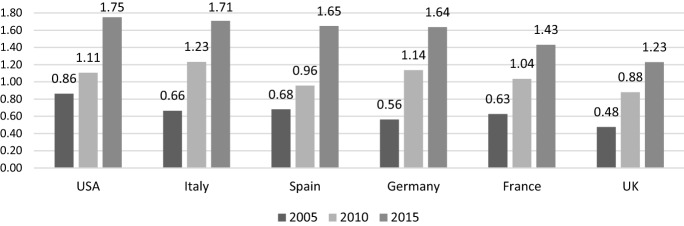
Backward participation (%) of selected countries with respect to China (2005, 2010, 2015). Source: authors’ elaboration on OECD-TiVA database

As stressed in the previous section, the strong interdependence of the economies has been crucial in spreading the economic contagion worldwide, following a domino scheme. In this context, the fact that China was the first economy affected by the COVID-19 represented a factor of paramount importance because of its central role in modern global production networks.^3^ An empirical illustration of this phenomenon is shown by Fig. 3, which reports the backward participation of prominent industrialized countries with respect to China for 2005, 2010 and 2015. This index is often used to approximate the level of international fragmentation of production, as it measures the percentage share of added value produced abroad (in China in this case) which is contained in the gross exports of each individual country over the total gross exports of the latter. In other words, this indicator measures to what extent the exports of countries under observation depend on the import of intermediate goods from China. As Fig. 3 shows, between 2005 and 2015 the backward participation index has more than doubled for all six countries, with the United States and Italy reaching the highest values in 2015, equal to 1.75% and 1.71% respectively. The integration of China into the global production networks of many other countries in the world follows a similar trend and in an even more accentuated way when looking at the remaining BRICS countries (Brazil, Russia, India and South Africa).

Focusing on Italy, it is worth mentioning that the industries showing the highest level of backward participation with respect to China report much higher and increasing values over time, which in 2015 spanned from 2.81% for machinery and equipment to 3.49% for textiles and wearing apparel sector, up to a level equal to 3.64% for computer and electronic and 4.33% for electrical equipment industry (data from OECD-TIVA).

Unsurprisingly, with the outbreak of the pandemic, this complex global interconnection of production—and the crucial role that the “factory of the world” plays in this context—has prompted part of the economic literature to warn about the need for many countries to promote supplier diversification with the aim of reducing their dependence on imports of intermediate goods from China (Javorcik 2020; Monga 2020).^4^

It is worth noting that, on the one hand, this diversification had been to a small extent already implemented by some MNCs, which had begun to diversify their supply sources in response to trade restrictions due to the trade war between the United States and China. In particular, some MNCs have moved production to other countries mainly in South East Asia and/or have undertaken reshoring policies—i.e. return of production back to the country of origin from overseas—a phenomenon we will briefly discuss below. On the other hand, it should not be forgotten that the breadth of the Chinese market makes the complete abandonment of the Asian giant very risky and unprofitable for many companies.^5^

However, the Italian backward linkages are not limited to Asian-centered production networks. Although the latter are gaining ever more prominence over time, the Italian industrial fabric shows a higher level of integration in European and North American GVCs (WTO 2019). Indeed, Italy reported an overall backward participation equal to 22.2% in 2015, of which the Chinese share accounted for less than 8% (data from OECD-TiVA). It follows that the success in fighting the contagion by China since March 2020 has only alleviated the GVCs-disruption burden on several Italian production activities. In fact, after being imposed in China, the lockdown hit even harder European economies and the US, with respect to which Italy shows an even stronger vertical integration.

**Fig. 4:**
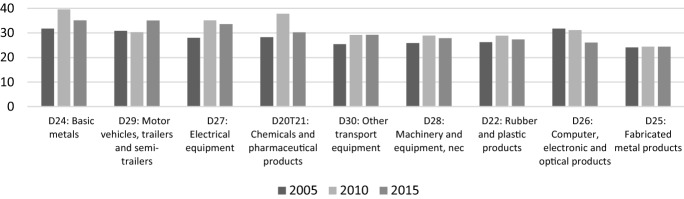
Backward participation (%) of Italian industries (2005, 2010, 2015). Source: authors’ elaboration on OECD-TiVA database

In this regard, Fig. 4 shows the Italian industries with the highest level of backward participation, i.e. those relying the most on foreign production, with the exclusion of the manufacturing industry of coke and refined petroleum products (whose backward index reaches almost 70% in 2015). The figure reports clear-cut evidence about the crucial role of imports from foreign suppliers for the production activity of key Italian industries such as basic metals, automotive, electrical equipment as well as chemicals and pharmaceutical products; for these sectors the amount of foreign value added the Italian firms process to export products to third countries is more than 30% of their gross exports. In other words, and beyond what happens in China, if the world stops because of COVID-19 pandemic, Italy can only suffer highly negative consequences, at least in the short period.

Notably, the evolution of the backward participation index of the Italian automotive industry (sector 29) is the one showing the most marked increase from 2005 to 2015, which goes from 30 to 35%. This growing trend mirrors the offshoring of manufacturing stages of production increasingly carried out by a share of Italian firms towards Central and Eastern European countries (CEECs) since the Nineties (Celi et al. 2018).

Furthermore, the growing vertical fragmentation of the automotive sector and the changing position of Italy in the global production network related to motor vehicles represents a good example to illustrate the potential implications of the pandemic on Italy’s economy.

**Fig. 5:**
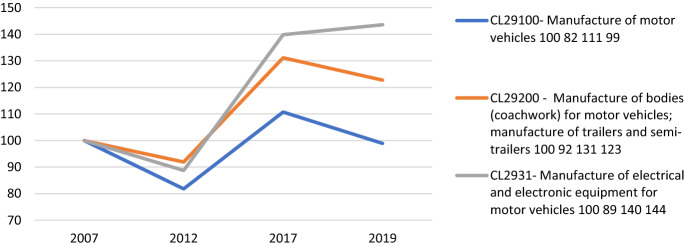
Volume of export of motor vehicles and components of motor vehicles of Italy, 2007–2019, 2007 = 100. Source: authors’ elaboration on ISTAT-Coeweb database

Especially after the Great Recession of 2008, the Italian automotive industry has clearly made a shift in GVCs, playing a greater role as a global supplier of components for car industry, while losing positions in the final markets of cars (Fig. 5). Although the car production is still important in the national economy, employing around 66,000 workers in 110 firms in 2017, it exhibits a lower export propensity than the car electrical components industry. In particular, after 2008, the latter largely increased its share of exports, shifting the functional role of the Italian automotive industry into GVCs and increasing the country’s specialization in the production of some parts of the automotive production chain, namely electrical components and bodies for motor vehicles. The traditional car industry, which used to be highly concentrated and dependent upon the Italian domestic market, has thus moved towards a specialization pattern more and more focused on the exports of motor vehicles components, while the national production of cars is stable if not declining (being its volume in 2019 equal to the one in 2007).

Hence, as the world stops because of pandemic, the Italian car industry suffers the interruption of purchases from foreign markets of its components while the home market has diminished its capacity to absorb them, due to the relatively low growth of its assembly activities. Quite symmetrically, the lockdown firstly imposed by Italian Government has represented inevitably a supply chain disruption also for the other countries relying on import of intermediate goods from Italy for production, exacerbating the economic crisis.

## Emerging trends and the role of industrial policy

Alongside the vulnerabilities of GVCs highlighted by the pandemic, in Italy the latter also showed how the austerity policies pursued in the last decade have made the healthcare system unable to successfully deal with emergencies (Prante et al. 2020). Although an ageing population requires larger health expenditures, the public health system shrank.^6^ Several healthcare profiles, like doctors and nurses, had to deal with a labour market with few professional opportunities and highly relying on temporary positions due to the outsourcing of health services to the private sector, especially in Lombardy—i.e. the Italian region most affected by COVID-19. The pandemic has thus revealed the shortage of health personnel, of medical infrastructures like Intensive Care Units (ICU) and of individual protection devices (IPDs), like face masks for the medical staff (Ranney et al. 2020).^7^

In this context, the pandemic caused a further reduction of the available medical staff due to a large amount of contagions because of the individual protection devices shortage, pushing the Italian government to set up a call to scale up the medical staff.^8^ In addition, the restriction on international trade and the export limits imposed by several Governments, aimed at ensuring national self-sufficiency, partially disrupted the supply chain of medical devices. Although the medical industry was not included into the lockdown policy due to its relevance for the emergency,^9^ offshoring of relevant segments of the manufacturing chain by Italian firms might have reduced the sourcing capacity of key medical equipment.

The pandemic also highlights how the extension of GVCs impacts on the “knowledge chain”. In fact, the COVID-19 emergency has shown that de-specializing in some key segments of healthcare equipment manufacturing is detrimental to the overall efficiency of the system and to its resilience in particular. For example, only a few Italian firms still have the knowledge to rapidly set up fully equipped ICUs if necessary, despite the long lasting Italian industrial tradition in the biomedical sector.

Another example is provided by the United Kingdom; the country’s long-term deindustrialisation process made it very difficult to cope with the pandemic. The Prime Minister Boris Johnson had to struggle to find national companies able to manufacture ICUs, leading him to negotiate with the automotive industry.^10^ At the same time, he faced a shortage of personal protective equipment and of health workers, who are essential for managing any ICU.^11^

The pandemic has thus highlighted the fragilities of the vertical disintegration of production across national borders, questioning at least partially the merits of fragmenting supply chains occurred in the last three decades. From this perspective, the global diffusion of COVID-19 is likely to contribute to a reduction of trade in GVC, a process that has already been recorded in the aftermath of the Great Recession of 2008, giving rise to a number of studies on production reshoring and back-shoring phenomena (Rodrik 2018).

There are several motivations underlying such a re-thinking of GVCs. First, a sluggish global demand and market saturation phenomena reduced the (domestic and cross-border) investment opportunities for firms. Second, China’s involvement in the global production networks appears to have decreased over the past decade—as documented by the sharp fall in backward participation of the country from 26.3% in 2005 to 17.3% in 2015 (data from OECD-TiVA). Third, reshoring and back-shoring strategies have become more common for MNCs, as a reaction to: fewer arbitrage opportunities on labour cost; higher coordination costs associated to global networking; and, last but not least, the diffusion of digital production technologies, making it convenient to re-internalize production without incurring in higher costs (Dachs and Zanker 2015; Dachs et al. 2017; ILO 2018; Seric and Winkler 2020). Alongside these factors, policy-driven anti-globalization forces as Brexit, trade wars and protectionist measure by the US have likely played a role (Dachs and Seric 2019).

In this scenario, the challenge for a new industrial policy concerns the capability to govern the “landslides” of a world economy constantly pressured by globalization and deglobalization forces. In other terms, leaving aside calls for hyper-globalization on one side and national self-sufficiency on the other, the mission of a modern industrial policy should be the consolidation of resilient GVCs on a reduced, largely regional, scale to pursue common development objectives.^12^

The strategy we are suggesting is not so different from what China is promoting. The Asian country in the last decade addressed huge public and private resources to diversify and upgrade its production matrix, moving from low- to high-value added segments of production while building an ever more complete value chain within the South-East Asian borders (European Union Chamber of Commerce in China 2017). This allowed China to partially shorten its GVCs and its dependence upon imports—especially for those inputs it strongly needed in the first times of its industrialisation—without giving up the productivity gains from specialization and trade (De Backer et al. 2018; Duan et al. 2018; Stollinger 2018).

In doing this, China has been building brand new production capacity with the aim of expanding and diversifying its productive matrix and enter in global markets from which Chinese firms were absent until a couple of decades ago. In front of this competitive challenge, the European economy needs to simultaneously combine greater resilience of the productive system to major disruptive events as pandemics, with a strengthened international competition of its firms on global markets. We suggest that this will inevitably require a vast investment plan at the continental level.

More precisely, we argue that a well-targeted European industrial policy aimed at consolidating its continental supply chains could take four birds with one stone.

First, aiming to fill in the gaps—even expanded by the pandemic—along its continental value chains, the European economy would enjoy the efficiency gains arising from the division of labour while fundamentally augmenting the resilience of its supply chains. This would be ever more important with regard to strategic productions, reason why Europe should act cooperatively sharing resources, knowledge and managing capabilities in industries like health, food supply, infrastructure, environmental and renewable energy and social security (see also Pianta et al. 2020).^13^ Moreover, the pandemic-induced reorganization of the global industrial structure could be an opportunity for the selection and evolution of small- and medium-size enterprises (Juergensen et al. 2020), also through their repositioning into shorter supply chains marked by lower power asymmetries.

Second, such European industrial plan could favour economic convergence among country members, finally closing the long-term structural asymmetries which triggered the European debt crisis of 2009–2013 and doomed Europe to a sluggish recover in the following years (Celi et al. 2018). It follows that investments needed to pursue such industrial strategy should be firstly addressed towards those European countries which in the last decades have suffered the most the progressive impoverishment of their production matrix (Celi et al. 2020; Landesmann and Stollinger 2018; Lucchese and Pianta 2020a). From this perspective, the consolidation of a European block of converging and well-integrated economies, effectively participating in regional level GVCs, could constitute a stronger basis to selectively develop intercontinental production networks. The latter could be aimed at gaining access to valuable resources and knowledge assets that might not be available and cost effectively developed within one region, and that can be conveniently sought elsewhere. European economies would then benefit from specialization and trade at a wider scale, while exerting a greater bargaining power in their effort to access such resources.

Third, while the hyper-specialization of national economies in a few phases of the value chain increases the mutual dependence of economies—up to exposing single countries to whatever shock occurring wherever in the global economy—it also risks to make it more difficult to convert domestic production in case of shortage of critical supplies. The industrial policy design we are advancing should aim to reduce such interdependence while increasing the responsiveness and adaptation of the productive system to both internal and external conditions.

Finally, an industrial policy aimed at strengthening the position of European national industries in the value chains of strategic productions could help reduce the social and economic costs and risks of hyper-specialization. In fact, a draw-back of the extreme fragmentation of production is that it has reduced the incentives of a part of firms to make investments for the development of those value chain activities—other than core competencies—which appeared too risky, not enough profitable, and largely offshorable (Antràs 2019; Dottling et al. 2017; Gutiérrez and Philippon 2016; Hummels and Klenow 2005). This has reduced their knowledge base while decreasing the resilience of European production networks. In fact, during the booming period of the international fragmentation of production and of greater expansion of GVCs, i.e. at the beginning of the XXI century with the entry of China in the global capitalistic market, the European economy witnessed a stagnant dynamics of investment in fixed assets, both tangible and intangible (Arrighetti 2020; Pisano and Shih 2012). The hyper-specialization of firms thus produced an incentive to outsourcing (and geographical dispersion of) the other value chain business activities (Baldwin and Gu 2009; Bernard et al. 2012). The consequence has been that also fixed capital investments have been concentrated on a increasingly narrow production matrix, causing a reduction of their productive capacity and of the pool of knowledge on which their ability to compete was built on. Such a dynamics points out a structural shortcoming in the process of cross-border vertical disintegration of production: not only key gaps in value chains involving strategic industries emerge, but the whole evolution of the technological matrix of European companies comes out depressed. Furthermore, all that goes hand in hand with stagnant productivity, weak employment dynamics and an impoverishment of the technological base of European companies vis-à-vis emerging companies overseas. This further clarifies the necessity and feasibility of a continental industrial policy plan aimed at recovering and expanding the technological matrix of companies and at the same time filling the gaps in the value chains involving strategic productions.

These considerations suggest that current tendencies of globalisation and GVCs should not be addressed with a “TINA” (There Is No Alternative) slogan; the same slogan which contributed to the acceleration of globalisation itself over the 1980s. Conversely, alternatives to overcome the weaknesses of modern global economy exist, as also mentioned in a World Investment Report of a few years ago: “Are active promotion of GVCs and GVC-led development strategies the only available options or are there alternatives? [The alternative] is an industrial development strategy aimed at building domestic productive capacity, including for exports, in all stages of production […] to develop a vertically integrated industry that remains relatively independent from the key actors of GVCs for its learning and upgrading processes.[…] It thus appears that countries can make a strategic choice whether to promote or not to promote GVC participation. To do so, they need to carefully weigh the pros and cons of GVC participation” (UNCTAD 2013, p. 175).

## Conclusions

In this contribution we illustrated the supply- and demand-side mechanisms through which the COVID-19 pandemic has triggered the economic contagion which led to a sharp reduction of production worldwide. We showed that between the pandemic and globalization a close and multi-faceted relationship exists. On the one hand, the outbreak of the pandemic has an impact on global production with a domino effect. On the other hand, global production has played a role in the amplification of the pandemic, especially accelerating its spread up to a rate never experienced before.

Further, we provided empirical evidence about the crucial role of GVCs in bringing about the leap forward in the level of interdependence of the economies on global scale. Most of this empirical evidence concerns the impact of the pandemic in Italy, one of the most affected countries. In this context, we underlined the weaknesses of Italian participation in GVCs as a pivotal factor in causing the collapse of production.

The debate about the future trend of international production and GVCs is open (EIU 2020; Lucchese and Pianta 2020b; Seric and Winkler 2020; Strange 2020). Many economists support the idea of an even increasing expansion of GVCs, as their widespread diversification might help reducing the global impact of national shocks (Baldwin and Evenett 2020; Baldwin and Weder di Mauro 2020; Monga 2020). However, the idea that “firms are eager, willing and able to meet any demand” (Baldwin 2020) proved to be at least over-optimistic. Conversely, one can quite convincingly argue that the pandemic has provided further reasons to rethink the pros and cons of GVCs, assessing the merits of cross-border production networks while highlighting the structural shortcomings of extreme specialization.

We suggest leaving aside calls for hyper-globalization on one side and national self-sufficiency on the other, arguing for a European industrial policy plan targeted to the consolidation of resilient GVCs on a continental scale to pursue common development goals. Such regional/continental level production networks—partially resembling attempts made by China to coordinate production within the South East Asian region—could provide a less fragile basis for the development of more selective, strategically oriented, intercontinental networks. At the same time, a European industrial policy which goes in this direction would significantly increase the growth rate of capital formation and expand the matrix of technology used, two tendencies which appear at odds with the productive hyper-specialization induced by the modern global fragmentation of production.

## Footnotes

1 We are grateful to Alessandro Arrighetti for the valuable comments on a previous version of this paper, which allowed to improve it considerably. The usual disclaimers apply.

2 See https://www.prometeia.it/atlante/produzione-industriale-marzo-2020

3 Notably, the Chinese city where the pandemic firstly outbreak, i.e. Wuhan, was a key production hub for both Chinese and world production, so much so that already in 2013 someone speculated that it might become “the Detroit of China” (“Can Wuhan Become the Detroit of China?”, *The Wall Street Journal*, 17 October 2013).

4 See also, among the others, “Samsung shifts some smartphone production to Vietnam due to coronavirus”, *Financial Times*, 6 March 2020; “Here's how global supply chains will change after COVID-19”, *World Economic Forum*, 6 May 2020; “US-China economic decoupling accelerates in first quarter of 2020”, *Financial Times*, 11 May 2020; “The Coming ‘Breaking’ Of The China Supply Chain”, *Forbes*, 17 May 2020.

5 “Reducing Global Supply Chain Reliance on China Won’t Be Easy”, *Brink News*, 22 April 2020.

6 See “Coronavirus cases are pushing Italy’s hospitals to the brink”, *Fortune*, 10 March 2020.

7 The Higher Education Reform (so-called Gelmini law) introduced important restrictions on access to university courses. In 2018 the first Conte government, in order to deal with an estimated shortage of 5,000 nurses and doctors in 2023 in the public health system, was therefore forced to remove some limitations on access to health specialization schools.

8 See “Italy will rush 10,000 student doctors into service, scrapping final exams”, *The Hill*, 18 March 2020.

9 Differently from the automotive industry whose production has fallen as a result of the lockdown policy in Italy and elsewhere, the Italian medical sector during the months of March and April increased its activities to deal with the domestic demand of healthcare units and individual protection devices.

10 See “Johnson’s Ventilator Plan Puzzles U.K. Firms Wanting to Help”, *Bloomberg*, 17 March 2020.

11 See “Coronavirus letter to the editor: Without protection, NHS staff are cannon fodder”, *The Times*, 22 March 2020.

12 Recent statements by the German and French finance ministers appear to be partially moving in this direction (see “Germany would like to localize supply chains, nationalization possible, minister says”, *Reuters*, 13 March 2020; “European industrial independence”, official site of the French government, 2 April 2020).

13 The Strategic Forum for Important Projects of Common European Interest (2019, p. 14) refers to “strategic autonomy in key value chains” at European level, showing to share at least in part the objectives we are suggesting.

## References

[CR1] Antràs, P. (2019). Conceptual aspects of global value chains, NBER Working Paper, no. 26539.

[CR2] Arrighetti, A. (2020). Crisi di offerta e crescita senza investimenti, siepi.org, https://siepi.org/wp-content/uploads/2020/05/Crisi-di-offerta-4-07052020.pdf. Accessed 30 June 2020.

[CR3] Baldwin J, Gu W, Dunne T, Bradford-Jensen J, Roberts MJ (2009). The impact of trade on plant scale, production-run length, and diversification. Producer dynamics: New evidence from micro data.

[CR4] Baldwin R, Elms DK, Low P (2013). Global supply chains: Why they emerged, why they matter, and where they are going. Global value chains in a changing world.

[CR5] Baldwin R (2016). The great convergence.

[CR6] Baldwin, R. (2020). The supply side matters: Guns versus butter, COVID-style. VoxEU.org, https://voxeu.org/article/supply-side-matters-guns-versus-butter-covid-style. Accessed 30 June 2020.

[CR7] Baldwin R, Evenett SJ (2020). COVID-19 and trade policy: Why turning inward won’t work.

[CR8] Baldwin R, Tomiura E, Baldwin R, Weder di Mauro B (2020). Thinking ahead about the trade impact of COVID-19. Economics in the time of COVID-19.

[CR9] Baldwin R, Weder di Mauro B (2020). Mitigating the COVID economic crisis: Act fast and do whatever it takes.

[CR10] Boehm CE, Flaaen A, Pandalai-Nayar N (2019). Input linkages and the transmission of shocks: Firm-level evidence from the 2011 Tōhoku earthquake. The Review of Economics and Statistics.

[CR11] Bonadio, B., Huo, Z., Levchenko, A. A., & Pandalai-Nayar, N. (2020). Global supply chains in the pandemic, NBER Working Paper, no. 27224. 10.1016/j.jinteco.2021.103534 PMC8633421 34866652

[CR12] Buckley PJ, Strange R (2015). The governance of the global factory: Location and control of world economic activity. Academy of Management Perspectives.

[CR13] Celi, G., Guarascio, D., & Simonazzi A. (2020). A fragile and divided European Union meets Covid-19: Further disintegration or ‘Hamiltonian moment’? *Journal of Industrial and Business Economics-Economia e Politica Industriale* (published in this Forum).

[CR14] Celi G, Ginzburg A, Guarascio D, Simonazzi A (2018). Crisis in the European Monetary Union: A core-periphery perspective.

[CR15] Dachs B, Kinkel S, Jäger A (2017). Bringing it all back home? Backshoring of manufacturing activities and the adoption of Industry 4.0 technologies. Journal of World Business.

[CR16] Dachs, B., & Seric, A. (2019). Industry 4.0 and the changing topography of global value chains, UNIDO Working Paper, no. 10.

[CR17] Dachs, B., & Zanker, C. (2015). Backshoring of production activities in European manufacturing, MPRA Paper No. 63868.

[CR18] De Backer K, De Lombaerde P, Iapadre L (2018). Analyzing global and regional value chains. International Economics.

[CR19] Dottling, R., Gutiérrez, G., & Philippon, T. (2017). Is there an investment gap in advanced economies? If so, why? In presented at the 2017 ECB Forum on Central Banking (Sintra). https://www.ecb.europa.eu/pub/conferences/shared/pdf/20170626_ecb_forum/T_Philippon_Is_there_an_investment_gap_in_advanced_economies_If_so_why_with_R_Dottling_and_G_Gutierrez.pdf. Accessed 30 June 2020.

[CR20] Duan Y, Dietzenbacher E, Jiang X, Chen X, Yang C (2018). Why has China’s vertical specialization declined?. Economic Systems Research.

[CR21] EIU 2020 is available here:https://www.eiu.com/n/campaigns/the-great-unwinding-covid-19-supply-chains-and-regional-blocs/. Accessed 30 June 2020.

[CR22] European Union Chamber of Commerce in China 2017 is available here: https://www.europeanchamber.com.cn/en/press-releases/2532. Accessed 30 June 2020.

[CR23] Gereffi G, Fernandez-Stark K (2016). Global value chain analysis: A primer.

[CR24] Gutiérrez, G., & Philippon, T. (2016). Investment-less Growth: An empirical investigation, NBER Working Paper, no. 22897.

[CR25] Hummels D, Klenow PJ (2005). The variety and quality of a Nation's exports. American Economic Review.

[CR26] ILO (2018). Robotics and reshoring.

[CR27] IMF 2020 is available here: https://www.imf.org/en/Publications/WEO/Issues/2020/04/14/weo-april-2020. Accessed 30 June 2020.

[CR28] Inoue H, Todo Y (2020). The propagation of the economic impact through supply chains: The case of a mega-city lockdown to contain the spread of Covid-19. Covid Economics-Vetted and Real-Time Papers.

[CR29] Javorcik B, Baldwin R, Evenett SJ (2020). Global supply chains will not be the same in the post-COVID-19 world. COVID-19 and trade policy: Why turning inward won’t work.

[CR30] Juergensen, J., Guimón, J., & Narula, R. (2020). European manufacturing SMEs amidst the COVID 19 crisis: Assessing impact and policy responses. *Journal of Industrial and Business Economics-Economia e Politica Industriale* (published in this Forum).

[CR31] Landesmann MA, Stöllinger R (2018). Structural Change, trade and global production networks: An ‘appropriate industrial policy’ for peripheral and catching-up economies. Structural Change and Economic Dynamics.

[CR32] Lucchese, M., & Pianta, M. (2020a). Europe’s alternative: A Green Industrial Policy for sustainability and convergence, MPRA Paper No. 98705.

[CR33] Lucchese M, Pianta M (2020). The coming coronavirus crisis: What can we learn?. Intereconomics.

[CR34] Monga, C. (2020). The misguided war on global value chains, *Project Syndicate.* https://www.project-syndicate.org/commentary/covid19-misguided-war-on-global-value-chains-by-celestin-monga-2020-05. Accessed 30 June 2020.

[CR35] Pianta M, Lucchese M, Nascia L (2020). The policy space for a novel industrial policy in Europe. Industrial and Corporate Change.

[CR36] Pisano GP, Shih WC (2012). Producing prosperity: Why America needs a manufacturing renaissance.

[CR37] Prante F, Bramucci A, Truger A (2020). Decades of tight fiscal policy have left the health care system in Italy ill-prepared to fight the COVID-19 outbreak. Intereconomics.

[CR38] Ranney ML, Griffeth V, Jha AK (2020). Critical supply shortages—the need for ventilators and personal protective equipment during the Covid-19 pandemic. New England Journal of Medicine.

[CR39] Rodrik, D. (2018). New technologies, global value chains, and the developing economies, pathway for prosperity commission background paper series, no. 1.

[CR40] Rothe C (2020). Transmission of 2019-nCoV infection from an asymptomatic contact in Germany. New England Journal of Medicine.

[CR41] Seric, A., & Winkler, D. (2020). COVID-19 could spur automation and reverse globalization—to some extent. VoxEU.org, https://voxeu.org/article/covid-19-could-spur-automation-and-reverse-globalisation-some-extent. Accessed 30 June 2020.

[CR42] Sforza A, Steininger M (2020). Globalization in the time of COVID-19. Covid Economics-Vetted and Real-Time Papers.

[CR43] Stollinger, R. (2018). Asian experiences with global and regional value chain integration and structural change, wiiw Research Report, no. 436.

[CR44] Strange, R. (2020). The 2020 Covid‑19 pandemic and global value chains. *Journal of Industrial and Business Economics-Economia e Politica Industriale* (published in this Forum).

[CR45] Strategic Forum for Important Projects of Common European Interest (2019). Strengthening Strategic Value Chains for a future-ready EU Industry. https://ec.europa.eu/docsroom/documents/37824. Accessed 30 June 2020.

[CR46] UNCTAD (2013). World investment report 2013. Global value chains: Investment and trade for development. United Nations, Geneva.

[CR47] UNCTAD (2020). The Coronavirus shock: A story of another global crisis foretold and what policymakers should be doing about it. In Trade and Development Report Update, 9 March.

[CR48] Wallace R (2016). Big farms make big flu: Dispatches on influenza, agribusiness, and the nature of science.

[CR49] World Bank (2020). World Development Report 2020: Trading for development in the age of global value chains.

[CR50] WTO (2019). Global value chain development report 2019: Technological innovation, supply chain trade, and workers in a globalized world.

[CR51] Zanfei, A., Coveri, A., & Pianta, M. (2019). FDI Patterns and Global Value Chains in the Digital Economy. In Working Papers Series in Economics, Mathematics and Statistics, University of Urbino, no. 3. https://ideas.repec.org/p/urb/wpaper/19_03.html

